# Fluoxetine prevents development of an early stress-related molecular signature in the rat infralimbic medial prefrontal cortex. Implications for depression?

**DOI:** 10.1186/1471-2202-13-125

**Published:** 2012-10-18

**Authors:** Rafael A Barreto, Frederick Rohan Walker, Peter R Dunkley, Trevor A Day, Doug W Smith

**Affiliations:** 1 School of Biomedical Sciences and Pharmacy Faculty of Health, University of Newcastle, Callaghan, NSW, 2308, Australia; 2 Priority Research Centre for Translational Neuroscience and Mental Health, University of Newcastle, Callaghan, NSW, 2308, Australia; 3 Hunter Medical Research Institute, John Hunter Hospital, New Lambton Heights, NSW, 2310, Australia; 4 Present Address Faculty of Science and Technology, Deacon University, Geelong, VIC, 3220, Australia

**Keywords:** Neurotrophin, Gene expression, Microrarray, Restraint stress, Signalling pathways

## Abstract

**Background:**

Psychological stress, particularly in chronic form, can lead to mood and cognitive dysfunction and is a major risk factor in the development of depressive states. How stress affects the brain to cause psychopathologies is incompletely understood. We sought to characterise potential depression related mechanisms by analysing gene expression and molecular pathways in the infralimbic medial prefrontal cortex (ILmPFC), following a repeated psychological stress paradigm. The ILmPFC is thought to be involved in the processing of emotionally contextual information and in orchestrating the related autonomic responses, and it is one of the brain regions implicated in both stress responses and depression.

**Results:**

Genome-wide microarray analysis of gene expression showed sub-chronic restraint stress resulted predominantly in a reduction in transcripts 24 hours after the last stress episode, with 239 genes significantly decreased, while just 24 genes had increased transcript abundance. Molecular pathway analysis using DAVID identified 8 pathways that were significantly enriched in the differentially expressed gene list, with genes belonging to the brain-derived neurotrophic factor – neurotrophin receptor tyrosine kinase 2 (BDNF-Ntrk2) pathway most enriched. Of the three intracellular signalling pathways that are downstream of Ntrk2, real-time quantitative PCR confirmed that only the PI3K-AKT-GSK3B and MAPK/ERK pathways were affected by sub-chronic stress, with the PLCγ pathway unaffected. Interestingly, chronic antidepressant treatment with the selective serotonin reuptake inhibitor, fluoxetine, prevented the stress-induced Ntrk2 and PI3K pathway changes, but it had no effect on the MAPK/ERK pathway.

**Conclusions:**

These findings indicate that abnormal BDNF-Ntrk2 signalling may manifest at a relatively early time point, and is consistent with a molecular signature of depression developing well before depression-like behaviours occur. Targeting this pathway prophylactically, particularly in depression-susceptible individuals, may be of therapeutic benefit.

## Background

Stress is a potent risk factor in the development of mood and anxiety disorders and other psychopathologies. For example, stress is an important non-genetic cause of major depressive disorder (MDD), with both acute and chronic forms capable of precipitating major depressive episodes [1,2]. A number of theories have been proposed to explain how stress alters brain structure and function in stress responsive areas [3] and there is compelling evidence for synaptic plasticity dysregulation, with much work having elucidated how the glucocorticoid and various neurotransmitter systems contribute to this dysregulation (for reviews see [4-9]). In order to better understand how stress affects brain function, we have previously used a chronic psychological stress model and found that this stress paradigm markedly upregulates deltaFosB expression, a marker of ongoing neuronal activity, in the infralimbic medial prefrontal cortex (ILmPFC) [10]. The ILmPFC is implicated in processing emotional context and, consistent with this notion, human patients with vmPFC lesions showed impaired social emotions [11]. Additionally, deep brain stimulation of the vmPFC region can prolong remission of depression in treatment-resistant patients, indicating a role for this brain region in depressive states [12,13]. These human data and our previous findings using chronic psychological stress, which is known to increase vulnerability to the development of depression-like symptoms [14], led us to initially focus on the ILmPFC to better understand the neurobiology of stress and how this might potentially lead to depression sequelae.

To identify as many ILmPFC mechanisms as possible that are involved in the response to repeated stress, we used a genome-wide, gene expression analysis approach. We also chose a restraint stress model as this type of psychological stress affords greater control in application of the stressor than with, for example, the social conflict model. As the neural correlates that underpin the transition to the depressive state are not understood, we tried to identify early stress-induced changes that may increase susceptibility to the development of a full depression-like state. It is known that multiple stress experiences are generally needed to cause MDD in humans and are absolutely required to develop depression-like symptoms in animals, so we used what can be considered a sub-chronic stress paradigm that does not lead to these behavioural symptoms. We found sub-chronic stress resulted in a molecular signature in the ILmPFC, specifically perturbed BDNF-Ntrk2 (note, Ntrk2 is also known as tyrosine kinase receptor type B, TrkB) signalling, that is consistent with known indices of the depressive state and that was prevented with fluoxetine treatment.

## Results

### Genome-wide gene expression analysis

Illumina RatRef-12 Expression BeadChip microarrays and GenomeStudio software were used for genome-wide screening. As gene expression changes are generally subtle in the brain [15], a relatively low fold-change cutoff of 1.2 was used. A statistical significance level of p≤0.05 was used after applying a false discovery rate (FDR) to correct for multiple testing [16]. Using these criteria, 263 genes were differentially expressed in the ILmPFC from the stress group compared to controls, with 24 genes having significantly higher expression levels and 239 with significantly lower expression levels (Table 1). The majority of differentially expressed genes showed a fold change of less than 3, consistent with the notion that brain gene expression changes are generally modest.

**Table 1:** Genes identified as differentially expressed by microarray analysis in the IL mPFC of rats submitted to sub-chronic restraint stress when compared to non-stress control (p≤0.05; 1.2 fold-change cut-off)

**Down-regulated genes**								**Up-regulated genes**	
MAP1B	−2.93	RGD1565549	−1.69	MLLT3	−1.55	RGD1306565	−1.44	LOC363380	1.92
BMPR2	−2.83	EEF2K	−1.69	RGD1561141	−1.54	RGD1565486	−1.44	LOC360941	1.76
LOC497804	−2.53	NRP1	−1.69	TRIO	−1.53	RGD1562123	−1.44	LOC501221	1.68
NTRK3	−2.28	KIF5C	−1.69	LOC362543	−1.53	CPD	−1.44	ALDH3B1	1.68
LOC497765	−2.24	LARP5	−1.68	LOC497681	−1.53	ASAH3L	−1.43	LOC498374	1.62
LOC309928	−2.13	RGD1307100	−1.68	GRIA3	−1.53	NCOA1	−1.43	LOC501224	1.58
MAP2	−2.09	RGD1564560	−1.68	LOC360990	−1.53	LOC291209	−1.42	LOC501093	1.55
HELZ	−2.09	TM9SF4	−1.68	GLG1	−1.53	ATRN	−1.42	LOC691487	1.54
RICS	−2.08	DSCAM	−1.68	RBBP6	−1.53	SRRM2	−1.42	LOC367381	1.49
ZFP537	−2.04	PRKCE	−1.68	LOC500867	−1.52	CDH9	−1.42	LOC691672	1.47
ODZ4	−2.00	RGD1563437	−1.67	MYT1L	−1.52	GPD2	−1.41	LOC501223	1.45
PCDH17	−1.95	USP45	−1.67	JMJD1C	−1.52	LOC362315	−1.41	RGD1561850	1.45
ODZ3	−1.95	RGD1308448	−1.66	UNC5C	−1.51	KPNB1	−1.41	GS3	1.44
RGD1566031	−1.95	RGD1307907	−1.66	SPON1	−1.51	WDFY1	−1.41	CLCC1	1.39
RIMS1	−1.93	ADD1	−1.65	LOC361942	−1.51	LOC498048	−1.41	LOC690672	1.39
ZFPM2	−1.93	CALN1	−1.65	VPS13D	−1.51	SIPA1L1	−1.41	LOC363434	1.39
ODZ3	−1.91	RGD1306245	−1.65	LOC302405	−1.51	RGD1306116	−1.41	LOC501245	1.39
AFF4	−1.89	MTMR9	−1.65	LOC363492	−1.51	ATF7IP	−1.40	LOC501089	1.39
LOC290704	−1.89	MYCL1	−1.64	RGD1566279	−1.50	NEO1	−1.40	LOC501399	1.38
APEG3	−1.88	KLF7	−1.64	LPHN1	−1.50	ABCA2	−1.40	RPL7	1.37
SGK	−1.85	RERE	−1.63	NTRK2	−1.50	CD47	−1.39	LOC691575	1.35
LOC361639	−1.85	RIMS2	−1.62	RGD1563873	−1.49	EHMT1	−1.39	LOC363320	1.35
ANK2	−1.84	TIMP2	−1.62	AKAP9	−1.49	SLC17A7	−1.39		
TNR	−1.83	SEMA6A	−1.61	LRP1	−1.48	NFIA	−1.38		
FALZ	−1.82	KCND2	−1.61	LOC500721	−1.48	RGS17	−1.38		
KCNC2	−1.80	LOC497770	−1.60	PIM3	−1.48	RGD1305534	−1.38		
LOC501548	−1.79	GSK3B	−1.60	LUC7L2	−1.47	ZFP148	−1.37		
CENTG1	−1.79	NFIX	−1.60	LOC497729	−1.47	CHD3	−1.36		
MDGA2	−1.78	GTF2IRD1	−1.60	OPCML	−1.46	LOC497754	−1.36		
TMOD2	−1.76	PDE10A	−1.59	NEGR1	−1.46	BPHL	−1.36		
LOC501637	−1.76	BRAF	−1.59	SLC1A2	−1.46	RELN	−1.36		
CRIM1	−1.75	SORL1	−1.59	NRIP1	−1.45	LOC362587	−1.35		
KLF5	−1.75	PUM1	−1.58	RGD1306101	−1.45	FAM108B1	−1.34		
C11ORF8H	−1.74	LCP1	−1.57	MYO5A	−1.45	USP2	−1.34		
SORCS1	−1.74	ZFP57	−1.57	ZDHHC13	−1.45	MAFG	−1.33		
EDNRB	−1.73	ARHGAP5	−1.57	PPFIA3	−1.45	ALCAM	−1.33		
TOB2	−1.73	LOC313658	−1.57	ATP2B3	−1.45	RGD1307284	−1.32		
SLC38A1	−1.72	CSPG4	−1.56	MAST1	−1.45	LOC363849	−1.32		
GABRB1	−1.72	LOC683578	−1.56	USP13	−1.45	CAMK2G	−1.32		
RGD1310722	−1.72	KIF5A	−1.56	LOC367779	−1.45	ATP6V0A1	−1.32		
PCDH19	−1.71	RGD1311049	−1.56	RGD1307696	−1.44	MPP6	−1.31		
CAMK2A	−1.71	RGD1308329	−1.56	CELSR2	−1.44	EML2	−1.31		
SORCS3	−1.71	SYNJ1	−1.55	DNAJC5	−1.44	LOC501145	−1.30		
DUSP8	−1.70	KIF1B	−1.55	EPHA5	−1.44				

### Pathways associated with stress-induced gene expression change

To better interpret the stress-induced changes in gene expression in the ILmPFC with regard to potential biological function, the list of differentially expressed genes was subjected to pathways analysis using the Database for Annotation, Visualisation and Integrated Discovery (DAVID) [17]. DAVID analyzes gene lists to statistically determine whether there is enrichment for genes that belong to *a priori* defined gene sets. Using the Kyoto Encyclopedia of Genes and Genomes (KEGG) - defined biological pathways, this analysis found enrichment in the list of genes for 8 pathways **(**Table 2**)**. Notably, neurotrophin signalling was the most significantly enriched pathway in the differentially expressed gene list. Two other pathways associated with neuroplasticity also had relatively high enrichment scores: long-term potentiation (LTP) and erbB signalling. Both of these pathways have been implicated in psychiatric disorders [7,9].

**Table 2:** Over-represented KEGG pathways in the ILmPFC, 24h post stress according to DAVID

**KEGG pathway**	pvalue*	**Fold Enrichment**	**Benjamini**	**FDR**	**Genes**
Neurotrophin signaling pathway (04722)	<0.001	6.239	0.006	0.078	BRAF, RGD1306565, CAMK2G, PTPN11, NTRK3, MAP3K5, GSK3B, NTRK2, PIK3CA, LOC685605, CAMK2A, LOC685653, LOC685626, LOC685590
ErbB signaling pathway (04012)	0.003	6.165	0.101	2.694	CBLB, BRAF, GSK3B, CAMK2G, PIK3CA, LOC685605, CAMK2A, LOC685626, LOC685653, LOC685590
Long-term potentiation (04720)	0.007	6.518	0.169	6.868	GRIN2B, BRAF, CAMK2G, GRIN2A, CAMK2A
Axon guidance (04360)	0.014	4.126	0.252	13.797	EPHA5, SEMA6A, NRP1, PLXNA2, GSK3B, UNC5C
Cell adhesion molecules (04514)	0.025	3.541	0.346	23.766	GLG1, NCAM1, ALCAM, NLGN2, NEO1, NEGR1
Amyotrophic lateral sclerosis (05014)	0.028	5.922	0.332	26.567	SLC1A2, MAP3K5, RGD1306565, GRIN2B, GRIN2A
Glioma (05214)	0.031	5.727	0.314	28.605	BRAF, CAMK2G, PIK3CA, LOC685605, CAMK2A, LOC685626, LOC685653, LOC685590
Phosphatidylinositol signaling system (04070)	0.044	4.991	0.375	38.162	SYNJ1, PIK3CA, LOC685605, LOC497978, LOC685626, LOC685653, PIP4K2B, LOC685590

* = p < 0.05 FDR = false discovery rate.

### Real-time quantitative PCR (RT-qPCR) confirmation of stress-induced gene changes

**Figure 1:**
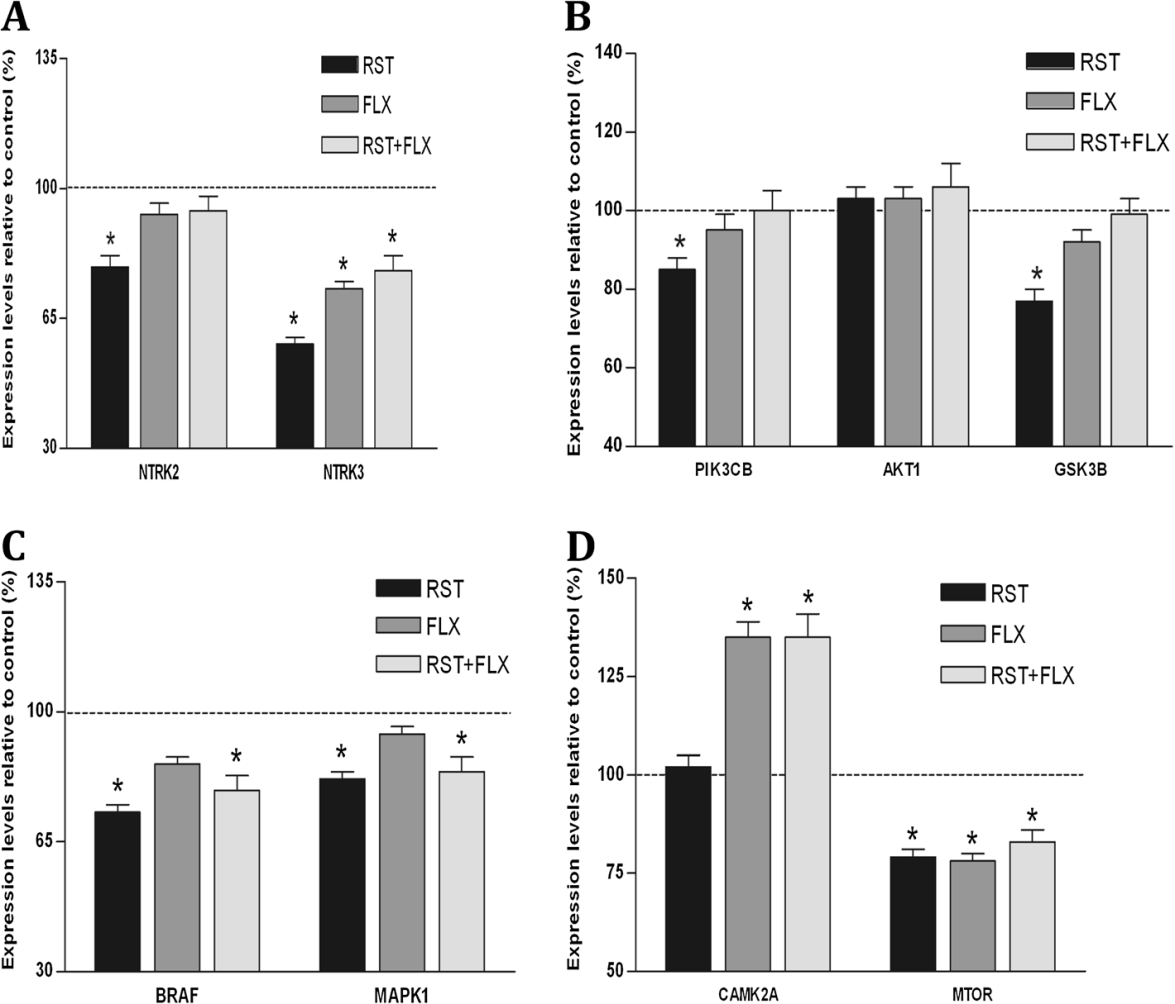
**A-D Graphs depicting the effects of sub-chronic restraint stress (RST), fluoxetine treatment without stress (FLX), and fluoxetine treatment with stress (RST+FLX) on gene expression in the ILmPFC.** Values are percentage means (±SEM) relative to unhandled controls (dashed horizontal line at 100%). Sub-chronic restraint stress reduced the transcript levels for the plasma membrane neurotrophin receptor genes ntrk2 and ntrk3 (**A**). Note, chronic fluoxetine treatment prevented the stress-induced ntrk2 but not ntrk3 transcript reduction. Sub-chronic restraint stress reduced transcript levels for the PI3K-AKT1-GSK3B (**B**) and NTRK2-MAPK/ERK (**C**) but not the PLCγ1 (**D**) intracellular signalling pathways. Interestingly, fluoxetine treatment prevented the sub-chronic stress-induced reduction in PI3K-AKT1-GSK3B signalling pathway, but not the NTRK2-MAPK/ERK (B-Raf and MAPK1 genes) pathway. Gene expression of the serine-threonine kinase, mTOR, a downstream target of the PI3K-AKT1 pathway, was reduced by sub-chronic stress, an effect not prevented by fluoxetine (**D**). * denotes p < 0.05.

In choosing specific genes for RT-qPCR confirmation, we used one or more of the following criteria: a) presence in enriched gene lists, as determined by DAVID analysis; b) a fold-change in either direction of ≥1.2, as determined by microarray analysis; c) experimental evidence in the literature supporting an involvement for the gene of interest in stress-related mechanisms. For instance, microarray and pathway analyses showed a down-regulation of genes encoding for the neurotrophin receptors *Ntrk3* (−2.28) and *Ntrk2* (−1.50) and other components of the neurotrophin (or Wnt) signalling pathway such as *Camk2a* (−1.71), *Gsk3β* (−1.60) and *Braf* (−1.59), in the ILmPFC of rats from the stress group. RT-qPCR analysis confirmed that the expression levels of the genes that encode for Braf, Gsk3β, Ntrk2 and Ntrk3 proteins were decreased in the ILmPFC of the stress group, in accordance with microarray results (Figure 1**)**. The decrease in *Camk2a* gene expression, however, was not confirmed by RT-qPCR. In addition, we probed other genes that were not identified by microarray analysis, but that we considered potentially important. RT-qPCR showed that expression of *Mapk1, mTOR* and *Pik3cb* genes were all significantly lower in the stressed group compared to controls. The expression level of *Akt1* was not significantly altered by stress.

### The effects of fluoxetine on ILmPFC stress-induced gene changes

As we found significant reduction in BDNF signalling-related genes in the stress group and perturbation in this pathway has been implicated in the aetiology of depression, we determined whether treatment with the antidepressant, fluoxetine, would alter the stress-induced changes in ILmPFC BDNF-related gene expression. As shown in Figure 1, fluoxetine treatment modulated the expression levels of genes involved in the neurotrophin signalling pathway. Fluoxetine significantly reduced the effect of stress on *Ntrk2, Gsk3β* and *Pik3cb* gene expression in the ILmPFC, such that the levels were not significantly different to home cage controls or fluoxetine treated controls. In contrast, fluoxetine did not alter the effects of stress on the expression of *Ntrk3*, *mTOR, Mapk1,* and *Braf* genes. Fluoxetine administration alone (fluoxetine control animals) also caused a significant decrease in expression levels of *Ntrk3* and *mTOR* when compared to controls without antidepressant or stress (home cage controls). Fluoxetine, and fluoxetine plus stress, caused significant and similar increases in *Camk2a* mRNA relative to controls and stress.

## Discussion

Stress is a potent risk factor for the development of depression, but the mechanisms that progress the brain’s normal response to stress to the pathological state that manifests as depression are poorly understood. Here, we have focussed on the ILmPFC to characterise gene expression changes following repeated, but sub-chronic, episodes of stress. We based our study design on the premise that early neurobiological indices of depression, or at least of the transition into a depression-like state, may be detectable in a sub-chronic model and we chose the PFC because of its known sensitivity to stress and its putative involvement in depression. For instance, stress causes dendritic remodelling in rat IL [18] and other mPFC regions [19,20], synaptic plasticity impairment [21], and deficits in PFC-mediated behaviours [22,23]. Consistent with these preclinical findings, MDD sufferers have reduced neuronal size [24], grey matter volume [25], and activity [26] in the subgenual PFC, the neuroanatomical equivalent of the rodent ILmPFC.

To observe depression-like behavioural and other changes in animals, it is necessary for the animal to experience repeated exposure to the stressor over prolonged periods. For example, in a systematic study of the effects of stress episode duration and number of repeats, Kim and Han demonstrated that at least 14 days of 2 hour daily restraint stress were required to produce significant depression-like behaviours [27]. Consistent with this finding, McLaughlin and co-workers found restraint stress of 6 hours per day for 21 days was required to induce morphological and functional changes in another brain region affected in depression, the hippocampus [28]. In a social interaction model, at least 10 consecutive days of social defeat appear necessary for depression-like symptomology to appear, at least in a subset of “susceptible” animals [14,29-31]. Similarly, multiple repeats of stress episodes are also necessary in the chronic mild stress model of depression [32]. It should be noted that single episodes of stress can elicit behavioural changes, however, these are considered characteristic of anxiety per se, rather than depression [33,34]. With respect to anxiety, we cannot completely discount the possibility that our sub-chronic stress-induced molecular changes are also related to anxiety. The link between stress and anxiety is well-established and there is considerable overlap in the putative mechanisms and behaviours between anxiety and depression. Furthermore, some antidepressants also have anxiolytic properties. Regarding this possibility, there is conflicting evidence in the literature. It is known that a single restraint episode, for example, can result in the delayed appearance of anxiety-like behaviour [35], and Kim and Han (2006) demonstrated that animals subjected to a more chronic restraint stress paradigm (6h/day for 10 consecutive days) did not display anxiety related behaviours as assessed with the elevated plus maze [27]. One explanation is that acute stress episodes are more likely to induce anxiety like behaviours and as the stress becomes more chronic there is a transition to a more depression-like behavioural phenotype. However, others have shown that anxiety-like behaviours can be displayed, in addition to depression-like ones, after many weeks of chronic restraint stress [36]. Clearly, more work needs to be done to understand the relationship between the nature of stress (type, intensity, duration, frequency) and the development of anxiety and depression. A potential limitation of the present study concerns the lack of a single acute stress group for comparison with the control and sub-chronic groups. Notionally, this type of acute stressor might have caused a similar molecular profile. However, we consider this possibility to be extremely unlikely given that Bland et al. (2007) used a single 80 minute session of electric shock, a stressor that is far more intense than our restraint stress, and found the stressed induced changes in neurotrophin transcripts in all regions of the mPFC, including ILmPFC, had returned to baseline by 24 hours post-stress [37].

To identify stress-related mechanisms that may potentially lead to the development of pathology, we first carried out a genome-wide gene expression analysis using moderately stringent criteria to define differentially expressed genes. A number of previous studies have also taken this approach [38], but direct gene-by-gene comparisons with these studies is problematic due to the different stress paradigms and microarray platforms used, as well as variation in brain region taken for analysis. Indeed, Surget and colleagues showed remarkably little overlap (zero genes for 3-region overlap) in gene expression between the cingulate cortex, amygdala and dentate gyrus of chronically stressed animals [39]. With regard the pattern of gene expression, we found the vast majority of differentially expressed genes were down-regulated, with most of the up-regulated transcripts being predicted genes or pseudogenes. Down regulation of a majority of genes in the PFC has also been observed following chronic stress [40,41]. To improve interpretability of the microarray data in relation to gene function in biological processes, we carried out pathway analysis whereby the degree to which sets of genes belonging to *a priori* determined biological pathways were enriched in the differentially expressed gene list was assessed using DAVID [17,42,43]. We identified eight significantly enriched pathways (Table 2), three had FDRs less than 10% and we focussed on these. Notably, all three of these pathways have been implicated in neuropsychiatric disorders [7,9].

The most significantly enriched pathway, and the one with the lowest FDR, was for neurotrophin signalling. There is a substantial body of evidence implicating BDNF in MDD. For example, BDNF and Ntrk2 transcripts and protein levels were markedly reduced in the PFC and amygdala of MDD subjects [44,45]. Also, serum BDNF is lower in MDD sufferers [46] and BDNF is being considered a predictive diagnostic marker for MDD [47]. Additionally, a number of chronic stress-based animal models of MDD have shown perturbation in the BDNF-Ntrk2 signalling pathway in various brain regions [48-50]. Indeed, Nestler and colleagues have demonstrated a crucial role for BDNF in the mesolimbic system for the development of depression-like behaviour [14,29]. We did not find stress-induced change in BDNF transcript levels in ILmPFC, which may imply that BDNF protein levels are also unchanged. This raises the question as to why Ntrk2 levels should change if BDNF does not. One possibility is that BDNF protein is delivered by dopaminergic afferents [14,29,51] to the ILmPFC and, consistent with this notion, we have found increased BDNF transcript levels in VTA dopamine neurons (manuscript in preparation). Alteration in the BDNF pathway can also be observed in some acute stress models, however as mentioned above, these changes are typically short-lived [37,52-55]. Our altered BDNF-Ntrk2 pathway finding was evident at 24 hours post the last stress episode of the sub-chronic stress paradigm and is consistent with a molecular signature of depression. The sub-chronic stress paradigm used in the present study does not induce depression-like behaviours, therefore, this molecular finding may represent an early mechanistic indicator of depression neuropathology. Prevention of the sub-chronic stress-induced BDNF-Ntrk2 perturbation by the antidepressant, fluoxetine, is consistent with this notion. Furthermore, it may also indicate that the ILmPFC is a particularly sensitive region to stress and therefore important in the development of the depressive state.

BDNF (or NT3/4) binding to Ntrk2 can activate three main intracellular signalling cascades: mitogen-associated protein kinase (MAPK; also known as the Ras/ERK-MAPK pathway), phosphotidylinositol-3 kinase (PI3K) - Akt, and the phospholipase C-γ1 (PLCγ1) cascades [56,57]. As Ntrk2 is the post-synaptic entry point to the BDNF/Ntrk2 signalling cascade, reduced ntrk2 levels might be expected to affect each of these intracellular signalling cascades and have significant impact on diverse cell functions. Indeed, genetically reduced BDNF/Ntrk2 levels result in altered activity-dependent synaptogenesis [58], synaptic function, such as LTP [59,60], learning [59] and, importantly, stress-related behaviours [61]. Our RT-qPCR data indicate that sub-chronic stress induced reduction in Ntrk2 transcript levels may in fact not impact each of these signalling pathways. For example, both the PI3K-Akt (PIK3CB and GSK3B genes) and MAPK/ERK (B-Raf and MAPK1 genes) pathways were affected by sub-chronic stress, but the PLCγ1 (CAMK2A gene) was spared. One important downstream target of the PI3K-Akt and MAPK/ERK pathways is the mammalian target of rapamycin (mTOR), a kinase involved in many cellular processes [62], including translation of synaptic proteins that underpin plasticity [63], and, therefore, potentially important in the development of the depressive state [9]. Although mTOR was not identified as being differentially expressed by microarray, using RT-qPCR we found it to be significantly reduced, consistent with changes in the aforementioned upstream regulatory pathways. This is supportive for a potential role in maladaptive neuronal plasticity contributing to the depressive state, although this will obviously require further experimental elucidation, particularly at the protein level.

Interestingly, antidepressant treatment with fluoxetine only prevented the sub-chronic stress-induced changes in the PI3K-Akt pathway related transcripts (PIK3CB and GSK3B genes), with B-RAF and MAPK1 gene transcripts of the MAPK/ERK pathway not significantly different to the stress without fluoxetine levels. This may indicate that the effects of fluoxetine antidepressant treatment are preferentially mediated by the PI3K-Akt pathway, at least in this brain region, and suggest that targeting components of this pathway may be of future therapeutic interest. In this regard it is notable that GSK3B has been implicated in the pathophysiology of mood disorders [8,64]. For example, GSK3B is one target for the mood-stabilising drug lithium, it is also required for the antidepressant effects of ketamine [65], and in MDD sufferers, GSK3B kinase activity is increased in the PFC [66]. Consistent with a role for GSK3B in depression, fluoxetine increases phosphorylation of GSK3B at a specific N-terminal serine residue, thereby decreasing the kinase’s activity [67]. Conversely, increasing GSK3B activity through viral-mediated overexpression, induces a depression-like phenotype in an animal model [68]. How a reduction in GSK3B at the transcript level, as seen in the present study, fits into this scheme is not clear. Interestingly though, and consistent with our data, GSK3B gene expression but not protein levels, was reduced in the nucleus accumbens of depression-susceptible animals in a chronic social defeat model of depression [68]. This similarity between our GSK3B finding and that of Wilkinson et al., 2011 [68], further supports our contention that we are seeing an early molecular signature of depression in the sub-chronic stress model. It should also be noted that GSK3B is not limited to the BDNF/Ntrk2 pathway as it is an important component of the Wnt-Frizzled signalling cascade, as well as being a downstream target of PKA, PKC and Akt [69], all of which are components of multiple signalling cascades. The Wnt-Frizzled signalling pathway has recently been implicated in depression [68,70].

We also found sub-chronic stress reduced ILmPFC transcript levels for Ntrk3, the cognate receptor for the neurotrophin, NT-3. Like BDNF, NT-3 has also been implicated in MDD. For instance, NT-3 gene expression was reduced in peripheral blood cells of individuals during depressive but not remissive states [71] and Ntrk3 transcript and protein levels were reduced in some brain regions of MDD patients [72] as were NT-3 levels [73]. Surprisingly, we found fluoxetine alone also reduced the expression of Ntrk3. Previously, it was shown that SSRI treatment had no effect on CSF NT-3 protein levels in MDD [74] and transcript levels in rat hippocampus [75]. The Ntrk3 transcript reduction seen in the present study may reflect a similar mechanism to that causing Ntrk2 reduction. Why this particular receptor was affected by fluoxetine remains to be determined.

## Conclusions

To further elucidate potential neurobiological mechanisms that may increase depression susceptibility, we have used a sub-chronic stress paradigm that is not capable of inducing behaviours that are characteristic of depression, and evaluated the infralimbic prefrontal cortex for molecular indices that may constitute an early signature for this psychopathology. We found that the BDNF-Ntrk2 pathway was affected by the stress paradigm as was GSK3B, which is a component of both the neurotrophin as well as the Wnt signalling cascades. Both the Ntrk2 and Wnt signalling pathways are implicated in depression and, consistent with the notion that our molecular findings represent early depression neuropathology, the stress-induced perturbations in these pathways were prevented by pretreatment with an antidepressant, fluoxetine, a selective serotonin reuptake inhibitor. These findings indicate that brain signalling pathways that are known to be abnormal in fully developed depressive states are actually perturbed well before the manifestation of behaviours that characterise depression.

## Methods

### Animals

Adult, male, Sprague–Dawley rats (20–22 weeks of age) were used for all experiments. Animals were obtained from the Animal Services Unit at the University of Newcastle, group-housed (4 per cage) on arrival and maintained in a temperature (21°C ± 1) and humidity controlled environment with food and water available *ad libitum*. Lighting was set for a 12:12 hour reverse light–dark cycle (lights off at 7:00AM; lights on at 7:00PM). All procedures were conducted in the dark phase. Animal housing and procedures were carried out in strict accordance with the University of Newcastle’s Animal Care and Ethics Committee regulations, the NSW Animal Research Act and Regulations, and the Australian Code of Practice for the care and use of animals for scientific purposes.

### Experimental procedures

*Stress* treatment: After a minimum of one week for acclimatisation, rats were randomly assigned to experimental groups (n = 6 per group) and housed 2 per cage. The control group consisted of animals that were maintained, without handling, under normal housing conditions until the day of sacrifice. We kept our control group as stress-naïve as possible in order to improve the probability of detecting subtle stress-related changes in the brain. It is known that handling alone is stressful to animals, although in the context of handling related stress and the mPFC, it has been shown that 7 days of handling rats for restraint stress did not alter mPFC pyramidal neuron dendrite morphology [20]. Animals in the stress group were subjected to a sub-chronic stress regimen that consisted of the handling necessary for and the daily sessions of 1 hour restraint in a Plexiglas tube (7.5 × 18 cm), for 5 consecutive days. There are many stress paradigms that can be used in pre-clinical studies of depression, however, they all suffer various limitations and there is no consensus as to which is the optimal one [76]. Restraint stress is a pain-free, physical stressor that elicits a stress response that is, in part, psychogenic, and one advantage of this stress method is the ability to readily control stressor parameters (intensity, duration, frequency), although, as with all stress paradigms, the individual animal’s stress response is not controllable. Importantly, we and others have shown chronic restraint stress can induce depression-like behaviours (e.g. anhedonia) and synaptic changes thought to contribute to these behaviours (e.g. dendritic retraction and impaired synaptic plasticity) [21,23,27]. Furthermore, the antidepressant fluoxetine has been shown to prevent development of depression-like behaviours following chronic restraint stress [77]. Taken together, these findings indicate the restraint stress model has a certain degree of construct, face and predictive validity [76] and is suitable for a sub-chronic exposure paradigm. The stress protocol was initiated at 10 a.m. each day and animals were returned to their pair-housed cage condition immediately after session completion. Animals were killed by an overdose of pentobarbitone (Lethabarb®) 24h after the last stress session. Food and water were available *ad libitum* in home cages.

*Chronic fluoxetine* treatment: Selective serotonin reuptake inhibitors (SSRIs) have been an important class of drugs for the treatment of depression ever since the introduction of fluoxetine, the original member of this class approved to treat humans. Much debate continues regarding the relative efficacies within and between the various classes of antidepressants, not to mention the specific molecular targets, intended and non-intended, of the antidepressants. Suffice to say, long-term treatment with SSRIs like fluoxetine, has been shown to significantly reduce the risk of relapse of depression [78] and, importantly, in the context of the present study, fluoxetine can prevent chronic stress induced brain BDNF deficits [50]. To assess the effect of fluoxetine antidepressant on ILmPFC gene expression following sub-chronic stress, a second batch of rats was randomly assigned into 2 groups (n = 6 per group) and in order to avoid injection stress, fluoxetine was administered via drinking water for 21 days prior to the start of the sub-chronic stress protocol. Rats were pair-housed to avoid social isolation stress. To deliver a target dose of approximately 10mg/Kg/day of fluoxetine hydrochloride ((7)-N-methyl-g-(4-[trifluoromethyl]-phenoxy)-benzenepropanamine), the average daily water intake was measured over a period of 4 days and the appropriate concentration of the drug then calculated to be delivered in that volume. Administration of fluoxetine at this dose, via drinking water to group-housed animals (up to 7 rats per cage), has been shown to be efficacious [79,80] and we have previously found this method results in a plasma fluoxetine concentration of 267 ±50 ng/ml (mean ± SEM; unpublished data). Body weight and drug solution intake were recorded daily throughout the period of fluoxetine administration**.** Fluoxetine treatment was maintained throughout the stress regimen until rats were killed at the end of the experiment. The fluoxetine control group received the same drug treatment but animals were maintained, without handling, under normal housing conditions until the day of sacrifice. Both groups had access only to fluoxetine-treated water for the duration of the experiment. Food was available *ad libitum.*

### Tissue preparation and RNA extraction

After decapitation and craniotomy, the brain was rapidly removed and cooled in ice-cold diethyl pyrocarbonate (DEPC)-treated PBS. Brains were then placed in an ice-cold metal brain matrix and the frontal lobe separated in the coronal plane and instantly frozen in dry-ice chilled isopentane. Tissue was stored at −80°C until needed. A series of 500 μm-thick coronal cryosections were obtained through the rostrocaudal extent of the ILmPFC (+2.5 to +4.0 mm relative to Bregma) [81]. Sections were then placed onto chilled RNAse-free glass microscope slides and the ILmPFC bilaterally excised using a 0.8 mm diameter stainless steel punch. ILmPFC tissue punches were obtained from three brain sections, pooled and homogenized with a motorized pestle in a RNAse-free microtube containing 350 μl of RNA lysis buffer (Qiagen). Homogenised samples were stored at −80°C until needed. Total RNA was then extracted and contaminating genomic DNA (gDNA) removed by in-solution DNase digestion followed by RNA Cleanup. RNA extraction, clean up and DNA digestion were done using RNeasy® Micro Kit and DNase reagents (Qiagen) according to manufacturer’s instructions. RNA yield and purity were estimated by absorbance spectrophotometry (NanoDrop 1000; Thermo Scientific). RNA integrity was evaluated by qPCR comparison of the relative levels of the 3^′^ and 5^′^ ends of the transcripts for the housekeeping genes glyceraldehyde-3-phosphate dehydrogenase (GAPDH) and β-actin (primers are listed in Table 3), as previously described [82]. RNA samples were further processed for either microarray hybridization or real-time, quantitative polymerase chain reaction (RT-qPCR).

**Table 3:** Primers used for validation of stress-responsive genes by RT-qPCR

**Gene ID**	**Symbol**	**Sense (5**^′^-**3**^′^**)**	**Antisense (5**^′^-**3**^′^**)**	**Amplicon size (bp)**
18S ribosomal RNA	*Rn18s*	CCCGAAGCGTTTACTTTGAA	CCCTCTTAATCATGGCCTCA	136
Glyceraldehyde-3-phosphate dehydrogenase	*Gapdh*	GAAGGGCTCATGACCACAGT	GGATGCAGGGATGATGTTCT	117
Actin, beta	*Actb*	CACACTGTGCCCATCTATGA	CCGATCGTGATGACCTGACC	272
Calcium/calmodulin-dependent protein kinase II alpha	*Camk2a*	GCCTGGACTTTCATCGATTC	GGTACTGAGTGATGCGGATGT	141
Glycogen synthase kinase 3 beta	*Gsk3β*	GCGAGACACACCTGCCCTCTTC	GTGGCCAGAGGTGGGTTACTTGAC	66
Mammalian target of rapamycin (serine/threonine kinase)	*mTOR*	TTGGATGTTCCAACCCAAGT	CAGGCCTTGGTTACCAGAAA	106
Neurotrophic tyrosine kinase, receptor, type 2	*Ntrk2*	TGGAGGGCGACCCACTCATCA	TCAGCTCGG TGGGCGGGTTA	123
Neurotrophic tyrosine kinase, receptor, type 3	*Ntrk3*	CATCCGCTGGATGCCACCTGAAA	AAGACACGGCCTTGGGTGATGCA	50
Phosphoinositide-3-kinase, catalytic, beta polypeptide	*Pik3cb*	CCTGCGACAGATGAGTGATG	CAATCCTCCGGTTGTCAAGT	134
V-raf murine sarcoma viral oncogene homolog B1	*Braf*	CATGGCGACGTGGCAGTGAAAATG	TGAGGTGTGGGTGCTGTCACATTC	50
Glyceraldehyde-3-phosphate dehydrogenase-3-prime	*Gapdh-3*^′^	GGCTGGCATTGCTCTCAA	GAGGTCCACCACCCTGTTG	88
Glyceraldehyde-3-phosphate dehydrogenase-5-prime	*Gapdh-5*^′^	GACAGCCGCATCTTCTTG	CACCGACCTTCACCATCTTG	63
Actin, beta-3-prime	*Actb-3*^′^	CCTAGCACCATGAAGATCAAGA	GCCAGGATAGAGCCACCAATC	77
Actin, beta-5-prime	*Actb-5*^′^	ACCCAGATCATGTTTGAGACCTT	CAGAGGCATACAGGGACAAC	79

### Gene expression microarray data processing

Microarrays were processed by the Australian Genomics Research Facility (AGRF; Melbourne, Australia). 100ng of total RNA, obtained from the ILmPFC was used for microarray analysis. For microarray analysis we used RNA from 4 of the 6 animals in each group, whereas for qPCR, RNA from each of the 6 animals was used. RNA quality was first checked using an Agilent Bionalyser, and then prepared for hybridization onto Illumina RatRef-12 Expression BeadChips. Arrays were scanned using standard Illumina protocols. RatRef-12 Expression microarrays probe for 21,910 genes. Raw intensity data was then imported into Illumina GenomeStudio Data Analysis Software and statistical analysis of gene expression was carried out using the Gene Expression module (version 1.1.1). First, a background measure based on the average signal of negative control probes was obtained and subtracted from all probes of the array. Next, the data was normalized by the GenomeStudio Average Normalization algorithm to adjust sample signals and minimize variation arising from non-biological factors. The p-values for differential expression were then calculated using the Illumina Custom Error Model algorithm and the Benjamini and Hochberg false discovery rate (FDR), a multiple testing correction method for adjustment of p-values [16]. Genes were considered to be differentially expressed if the comparison resulted in a p-value ≤ 0.05 and a ≥ 1.2 fold-change in expression (in either direction).

### Real-time quantitative polymerase chain reaction (RT-qPCR)

For confirmation of gene expression changes, RT-qPCR was carried out on mRNA from all 6 animals of each group. Gene-specific primer pairs (Table 3) were designed with the web-based NCBI primer-BLAST software ( http://www.ncbi.nlm.nih.gov/tools/primer-blast/), and targeted sequence near the 3^′^ end of the cDNA and, where possible, amplicons spanned intron:exon boundaries. cDNA was generated by reverse transcription using SuperScript III (Invitrogen) according to manufacturer’s instructions. Briefly, 200ng of total RNA, 1μl of 50μM) oligo(dT)_20_ primer, 0.5μl of 20μM 18S RNA-specific primer (5^′^-GAACTACGACGGTATCTGA-3^′^), 1μl of 10mM dNTP, and molecular biology grade water to 13μl, were mixed and heated for 5 minutes at 65°C, then chilled on ice for 1 minute. 4μl 5X First-Strand Buffer, 1μl of 0.1M DTT, 1μl RNaseOUT (40 units/μl) and 1μl SuperScript III RT (200 units/μl) were added and the mixture incubated for 60 minutes at 50°C, followed by 15 minutes at 70°C. Reverse transcription reactions without reverse transcriptase were also done to assess gDNA contamination. qPCR reactions were carried out in 12μl volumes containing: 6μl 2X SensiMixPlus SYBR (Quantace); 200nM each of forward and reverse primers, except for 18S rRNA, where 1μM was used; 1ng cDNA; molecular biology grade water to 12μl. After an initial 10 minute, 95°C enzyme activation step, 40 cycles of 95°C for 30 sec (step 1) followed by 60°C for 31 sec (step 2) were completed. Only primers that produced a single amplified product as shown by melt curve and gel electrophoresis analyses were used. Reactions were carried out on an ABI 7500 Real-Time PCR System (Applied Biosystems) and analyzed using the Applied Biosystems 7500 Sequence Detection Software (Version 1.4). Relative expression levels were determined using the comparative Ct method (ΔΔCt; [83], where gene expression was first normalised to the average of 18S rRNA, GAPDH and β-actin, to generate a ΔCt for each gene and sample, and then the average ΔCts for each gene were compared between the various groups. Statistical analysis of relative gene expression was done by comparisons between groups using Student’s t tests with Bonferroni correction to the alpha level to control for family-wise error.

### Pathway analysis

Pathway analysis of differentially expressed genes was undertaken using the Database for Annotation, Visualization, and Integrated Discovery bioinformatics resource (DAVID; http://david.abcc.ncifcrf.gov/; [17]. DAVID is a gene-centered database that integrates gene annotation resources and facilitates high-throughput gene functional analysis by conferring biological meaning to a gene. Enrichment analysis was performed in the “Pathway” annotation category primarily using the Kyoto Encyclopedia of Genes and Genomes (KEGG) pathway database for annotated terms. All other analysis parameters were left as the default. For a detailed description of the algorithms and statistical parameters used by DAVID for enrichment results see [42].

## Abbreviations

ILmPFC: Infralimbic medial prefrontal cortex; BDNF: Brain-derived neurotrophic factor; ntrk2: Neurotrophin receptor tyrosine kinase 2; MDD: Major depressive disorder; vmPFC: Ventromedial PFC; FDR: False discovery rate; DAVID: Database for annotation, visualisation, and integrated discovery; KEGG: Kyoto encyclopedia of genes and genomes; LTP: Long-term potentiation; RT-qPCR: Real-time quantitative PCR; mRNA: Messenger RNA; MAPK: Mitogen-associated protein kinase; PI3K: Phosphotidylinositol-3 kinase; PLCγ1: Phospholipase C-γ1; mTOR: Mammalian target of rapamycin; GSK3B: Glycogen synthase kinase 3B; PKA: Protein kinase A; PKC: Protein kinase C; Akt: Also known as protein kinase B (PKB); DEPC: Diethyl pyrocarbonate; RNA: Ribonucleic acid; gDNA: Genomic DNA; GAPDH: Glyceraldehyde-3-phosphate dehydrogenase; AGRF: Australian genomics research facility; cDNA: Complementary DNA; dNTP: Deoxy nucleotide triphosphate; DTT: Dithiothreitol.

## Competing interests

The authors declare they have no competing interests.

## Authors’ contributions

RB carried out all the experimental work, performed the pathways and qPCR statistical analyses, prepared all figures and tables and assisted with the writing of the manuscript. FRW contributed to the conceptual design of the study and assisted with critical revisions of the manuscript. PRD and TAD contributed to data interpretation and critical revisions of the manuscript. DWS was responsible for overall design and execution of the study, analysis and interpretation of the data and writing of the manuscript. All authors read and approved the final manuscript.
